# High‐Resolution Heterogeneous Hydrogel Printing Using a Home Projector

**DOI:** 10.1002/smtd.202500631

**Published:** 2025-06-23

**Authors:** Zhangkang Li, Jaemyung Shin, Kartikeya Dixit, Daichen Liu, Hongguang Zhang, Qingye Lu, Hitendra Kumar, Keekyoung Kim, Jinguang Hu

**Affiliations:** ^1^ Basic Medical Research Center Medical School of Nantong University Co‐Innovation Center of Neuroregeneration Nantong Jiangsu 226001 China; ^2^ Department of Biomedical Engineering University of Calgary 2500 University Drive, NW Calgary Alberta T2N 1N4 Canada; ^3^ Department of Mechanical and Manufacturing Engineering University of Calgary 2500 University Drive, NW Calgary Alberta T2N 1N4 Canada; ^4^ Department of Chemical Engineering University of Waterloo 200 University Avenue West Waterloo Ontario N2L 3G1 Canada; ^5^ Department of Chemical and Petroleum Engineering University of Calgary 2500 University Drive NW Calgary Alberta T2N 1N4 Canada; ^6^ Department of Biosciences and Biomedical Engineering Indian Institute of Technology Indore Indore Madhya Pradesh 453552 India

**Keywords:** 4D printing, cell alignment, cell organization, heterogeneous hydrogel printing, pattern encryption

## Abstract

Soft hydrogels are being increasingly recognized for their versatility and unique properties, making them attractive for a range of applications in tissue engineering, biomedical devices, and beyond. Among fabrication methods, 3D printing stands out for its precise control over material distribution, enabling the creation of complex structures. Traditional printing methods, however, struggle to produce heterogeneous hydrogels with diverse properties. Here, a novel approach is introduced utilizing polyvinyl alcohol bearing styrylpyridinium groups (PVA‐SbQ) for high‐resolution heterogeneous hydrogel printing. By leveraging the photoreactive nature of PVA‐SbQ, precise control over crosslinking time at different positions within a PVA‐SbQ hydrogel is demonstrated using a simple home projector. This enables the creation of intricate patterns with tailored properties within a heterogeneous hydrogel, showcasing synergistic combinations of soft and tough domains, as well as high and low swelling regions. The method not only advances the field of hydrogel printing but also holds promise for applications in pattern encryption, 4D printing, cell organization, and cell alignment. By overcoming the limitations of traditional printing techniques, the approach opens new avenues for the fabrication of complex and heterogeneous hydrogel structures with diverse applications in biomedical engineering and beyond.

## Graphical abstract

This study presents a high‐resolution hydrogel printing method using photoreactive polyvinyl alcohol bearing styrylpyridinium groups (PVA‐SbQ) and a standard digital projector to spatially control light exposure. This approach enables precise tuning of crosslinking, allowing the integration of soft/tough and high/low swelling regions within a single construct for advanced biomedical and responsive material applications.

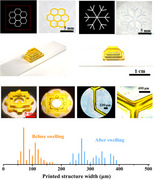

## 1 Introduction

Soft hydrogels have emerged as a versatile class of materials with unique properties that make them particularly appealing for a wide array of applications, ranging from tissue engineering to biomedical devices and beyond due to their hydrophilicity and 3D structures.^[^ ^1^, ^2^, ^3^, ^4^ ^]^ Among the various methods of fabrication, 3D printing has garnered significant attention due to its ability to precisely control the spatial distribution of materials, enabling the creation of complex hydrogel structures with tailored properties.^[^ ^5^, ^6^, ^7^ ^]^ In recent years, the development of hydrogel printing techniques has opened up new avenues for research and innovation in various fields, including biomedicine,^[^ ^8^ ^]^ tissue engineering,^[^ ^9^ ^]^ drug delivery,^[^ ^10^ ^]^ and advanced manufacturing.^[^ ^11^ ^]^ For example, a study conducted by Lee et al. demonstrated the fabrication of complex vascular networks using hydrogel printing, offering potential applications in organ‐on‐a‐chip systems and regenerative medicine.^[^ ^12^ ^]^ Additionally, another study led by Wei et al. investigated the use of hydrogel printing for the controlled release of therapeutic agents, highlighting its utility in precise drug delivery systems with customizable release profiles.^[^ ^13^ ^]^ Traditional hydrogel printing methods often involve techniques such as extrusion‐based printing,^[^ ^14^, ^15^ ^]^ inkjet printing,^[^ ^16^, ^17^ ^]^ and stereolithography (SLA) printing.^[^ ^18^, ^19^ ^]^ These printing methods typically necessitate a compatible printer and appropriate bioink, limiting them to the production of homogeneous hydrogels with a single set of properties. However, as biomedicine and biofabrication progress, the necessity arises to print intricate hydrogels with diverse properties distributed across multiple locations. Accordingly, the printing of isotropic heterogeneous hydrogels with defined patterns remains difficult.^[^ ^20^ ^]^ Additionally, high‐resolution printing is indispensable for heterogeneous hydrogel printing as it enables precise control over material deposition, fine feature reproduction, controlled crosslinking, and the creation of functional gradients within the printed structures.^[^ ^21^ ^]^

Polyvinyl alcohol bearing styrylpyridinium groups (PVA‐SbQ), prepared by grafting styrylpyridinium groups onto polyvinyl alcohol polymer chains, demonstrate exceptional crosslinking performance and hydrogel properties when utilized in the creation of multifunctional hydrogels under light exposure.^[^ ^7^, ^22^, ^23^ ^]^ This polymer amalgamates the inherent characteristics of PVA, such as biocompatibility and hydrophilicity, with the functional attributes of the styrylpyridinium group, including photoactivity and a cationic group. The photoreactive nature of styrylpyridinium groups enables the photo‐crosslinking of the polymer, facilitating the fabrication of 3D hydrogel structures with adjustable mechanical properties. Notably, the printing process necessitates no toxic crosslinkers or photoinitiators, resulting in enhanced cell viability and proliferation. In previous explorations, PVA‐SbQ has been proven to serve as a bioink for laser direct writing printing due to its distinctive rapid photodimerization characteristics.^[^ ^7^ ^]^ Additionally, the homogeneous properties of a PVA‐SbQ hydrogel, such as mechanical strength, swelling capacity, and adhesive properties, can be readily adjusted by altering the SbQ double‐bond crosslinking density through modulation of light irradiation time.^[^ ^7^ ^]^ However, establishing diverse hydrogel properties in distinct locations within a heterogeneous hydrogel remains a significant challenge.

Currently, achieving precise control over the printing of heterogeneous hydrogels demands meticulous attention to material selection, printing setup, design, process parameters, and post‐processing steps to attain the desired structure with customized properties across different regions.^[^ ^24^, ^25^ ^]^ Leveraging the unique crosslinking performance of PVA‐SbQ, our proposed method offered a streamlined and adaptable approach to hydrogel printing. In this manuscript, we presented a comprehensive exploration of crosslinking time control for fabricating high‐resolution heterogeneous hydrogel structures. Through the utilization of a simple home projector, we systematically manipulated the crosslinking time at various spatial locations, showcasing our ability to create intricate patterns of PVA‐SbQ hydrogels. These patterns exhibited synergistic combinations of soft and tough domains, as well as high and low swelling regions. Our approach not only introduced a novel method for achieving high‐resolution heterogeneous hydrogel printing but also holds significant promise for applications in pattern encryption, 4D printing, and cell organization. Overall, our findings underscored the potential of employing crosslinking time control with a home projector as a potent tool for designing and fabricating high‐resolution heterogeneous hydrogel structures with tailored properties. This methodology opened up a multitude of possibilities across diverse disciplines, from advanced materials science to biomedical engineering, offering both fundamental insights and practical applications.

## 2 Results and Discussion

### 2.1 Hydrogel Crosslinking

**Figure 1:**
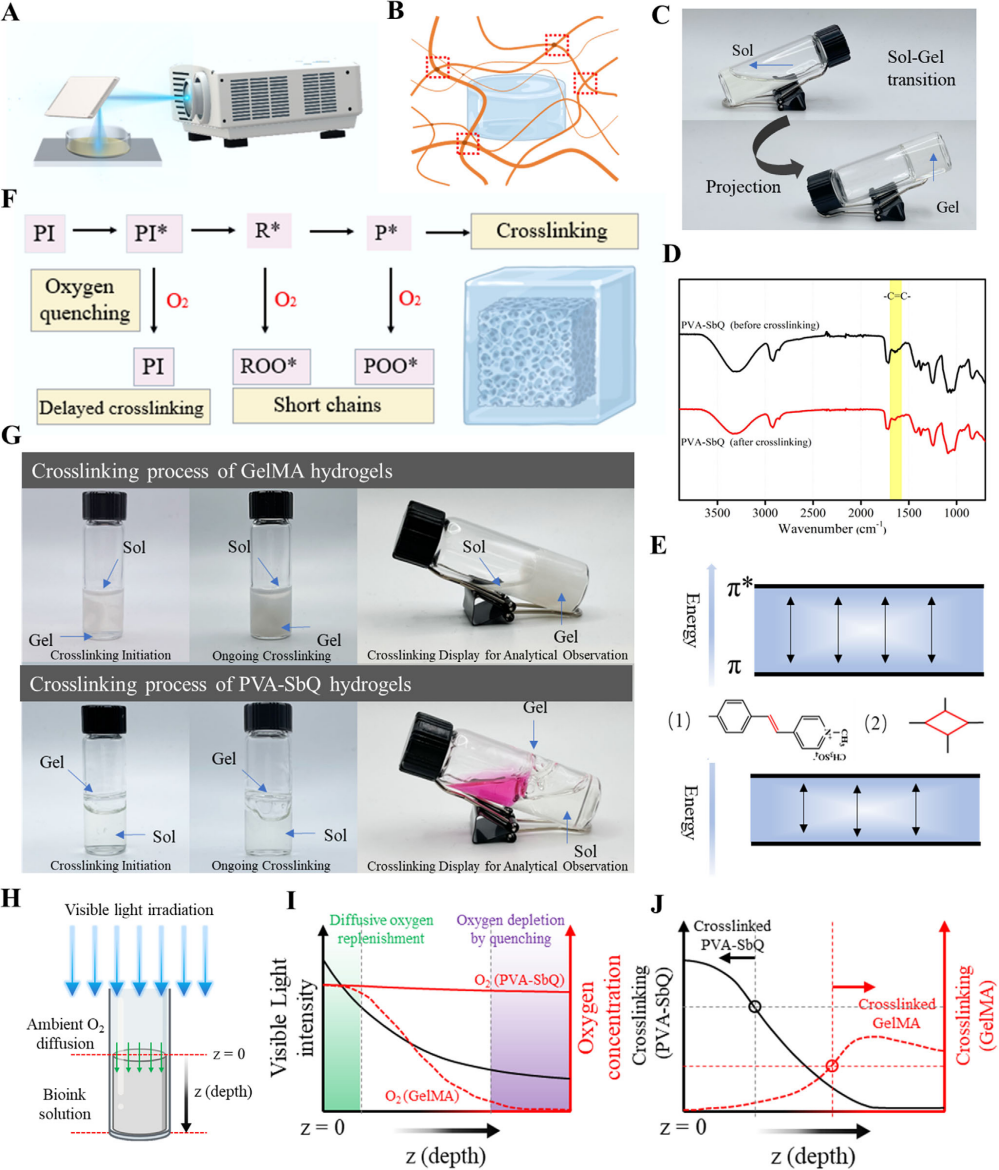
A) The schematic representation of the projector and printing method. B) the sketch of the crosslinked hydrogel structure. C) The sol‐gel transition process of PVA‐SbQ. D) The infrared spectra of uncrosslinked PVA‐SbQ and crosslinked PVA‐SbQ hydrogels. F) The negative effects of oxygen on crosslinking. G) The crosslinking process of GelMA and PVA‐SbQ hydrogels. H) The schematic diagram of hydrogel crosslinking under the visible light irradiation generated by a projector. I) The changes in visible light intensity and oxygen concentrations with the increase in the depth. J) The crosslinking of PVA‐SbQ and GelMA inks at various depths.

Previous studies demonstrated that high concentration PVA‐SbQ (8‐13%) solution could quickly transition from sol to gel under UV light and laser beam.^[^ ^7^, ^22^ ^]^ The light intensity of UV and laser beams is much higher than that of a home project. In order to assess the printability of PVA‐SbQ hydrogel under low light intensity from a projector lamp, the initial investigation in this study delved into the crosslinking process of hydrogel precursors at low concentrations, while concurrently examining the associated crosslinking mechanism. In this paper, 4% PVA‐SbQ solution was irradiated by a visible light projector instead of UV light or laser beam (**Figure** **1**A). During this process, PVA polymer chains gradually connected with the time due to the existence of photoreactive SbQ groups (Figure 1B), which resulted in sol‐gel transition (Figure 1C). Most photo‐crosslinked hydrogels require UV light illumination and/or the addition of crosslinkers and photo‐initiators.^[^ ^26^ ^]^ On the contrary, the SbQ groups triggered fast chemical crosslinking under light from a projector without the addition of chemical crosslinkers and photo‐initiators. The outstanding crosslinking ability of PVA‐SbQ under visible light was owed to its unique structure (Figure S1, Supporting Information). In typical photo‐crosslinking reactions, the formation of crosslinking is attributed to the polymerization of carbon‐carbon double bonds.^[^ ^27^ ^]^ The crosslinking of PVA‐SbQ also followed this principle. As depicted in Figure 1D, the peak area corresponding to the carbon‐carbon double bond diminished after crosslinking, indicating a reduction in the double bond concentration during the sol‐gel transition. However, conventional photoreactive polymers containing double bonds require photoinitiators to form hydrogels through light‐induced crosslinking (Figure S2, Supporting Information). When exposed to ultraviolet (UV) light or visible light, a photoinitiator generates free radicals, which initiate the opening of the double bonds in the polymer chains. These opened double bonds act as crosslinking points, reacting with other double bonds and forming a network of crosslinked polymer chains. This network traps water molecules, resulting in a hydrogel. The process creates a 3D, water‐swollen structure with enhanced mechanical and chemical stability due to the crosslinking at the double bond sites. Unlike the photo‐crosslinking of other polymers (Figure S2, Supporting Information), in PVA‐SbQ, four‐membered rings formed instead of crosslinking points due to their conjugated structure (Figure S1, Supporting Information). The conjugated structure of SbQ, along with the formation of four‐membered rings, led to a reduction in the gap between π orbital and π^*^ orbital. Consequently, this decreased the energy required for electron transition (Figure 1E). As a result, PVA‐SbQ could be crosslinked using low‐intensity visible light, such as that emitted by a home projector.

Furthermore, no photoinitiators were required in the photocrosslinking process, which ensured that the crosslinking of PVA‐SbQ remained unaffected by oxygen. In typical radical mediated polymerization processes, the inhibition by oxygen occurs because the radicals generated by photoinitiators can react with molecular oxygen (O_2_) to form peroxide radicals (O_2_^−^•), a process known as oxygen quenching (Figure 1F).^[^ ^28^, ^29^, ^30^ ^]^ These peroxide radicals can then deactivate photoinitiator radicals, preventing them from initiating polymerization reactions efficiently. Additionally, Oxygen can interfere with free radical polymerization reactions by reacting with free radicals such as reactive radical species (R^*^), and the polymer chain ends with an active site (P^*^), thereby prematurely terminating polymerization. This interference often leads to the formation of shorter polymer chains, ultimately resulting in a decrease in mechanical properties such as strength and elasticity. We demonstrated these phenomena by comparing photocrosslinking of Gelatin Methacryloyl (GelMA) and PVA‐SbQ. Being a radical driven process, the crosslinking of GelMA triggered by a photoinitiator did not initiate from the side closest to the light due to air interference, whereas PVA‐SbQ remained unaffected by air and started crosslinking from the area nearest to the light source. As shown in Figure 1G and Movies S1 and S2 (Supporting Information), GelMA underwent gel formation from the middle, while the surface layer, exposed to oxygen, remained in a liquid state. In contrast, PVA‐SbQ hydrogels initiated crosslinking from the surface and were unaffected by oxygen. To provide a more intuitive illustration, red dyes were introduced into the mixture but did not blend with the liquid below due to the presence of crosslinked hydrogel on the surface. The comparison of oxygen inhibition in radical mediated crosslinking and PVA‐SbQ is illustrated by considering visible light irradiation on a glass vial filled with the prepolymer solution and exposed to an ambient environment (Figure 1H). The variation of light intensity along the depth of the pre‐polymer solution due to the attenuation explained by Beer‐Lambert law is shown in Figure 1I. A previous simulation study of radical mediated photocrosslinking system described that both light intensity and oxygen concentration decreased progressively with depth (Figure 1I).^[^ ^30^ ^]^ Since the prepolymer solution was exposed to an ambient environment, there was an influx of oxygen from the exposed surface which subsequently diffused to the lower portion of the prepolymer solution. This diffusive transport was driven by the oxygen depleted region formed toward the bottom of the prepolymer solution layer due to the consumption of oxygen in the radical quenching process. For PVA‐SbQ hydrogels, which were not affected by oxygen, a very low concentration gradient of oxygen was observed within the prepolymer solution. Further, the highest degree of crosslinking occured at Z = 0 due to the highest light intensity at the beginning and the absence of quenching processes. Conversely, GelMA hydrogels were heavily influenced by oxygen, preventing crosslinking at the Z = 0 position. Taking into account the impact of light intensity as well as the low concentration of oxygen, the highest degree of crosslinking of GelMA hydrogel was observed in the middle section when GelMA started crosslinking (Figure 1J). Moreover, the crosslinking process of PVA‐SbQ, which didn't require the use of additional photoinitiators and relied on mild visible light for hydrogel preparation, exhibited low cytotoxicity.^[^ ^7^, ^31^, ^32^ ^]^ This property enabled the use of the hydrogel as a biocompatible material platform for cell organization and alignment studies conducted in the later sections of this work.

### 2.2 Hydrogel Properties Characterization

**Figure 2:**
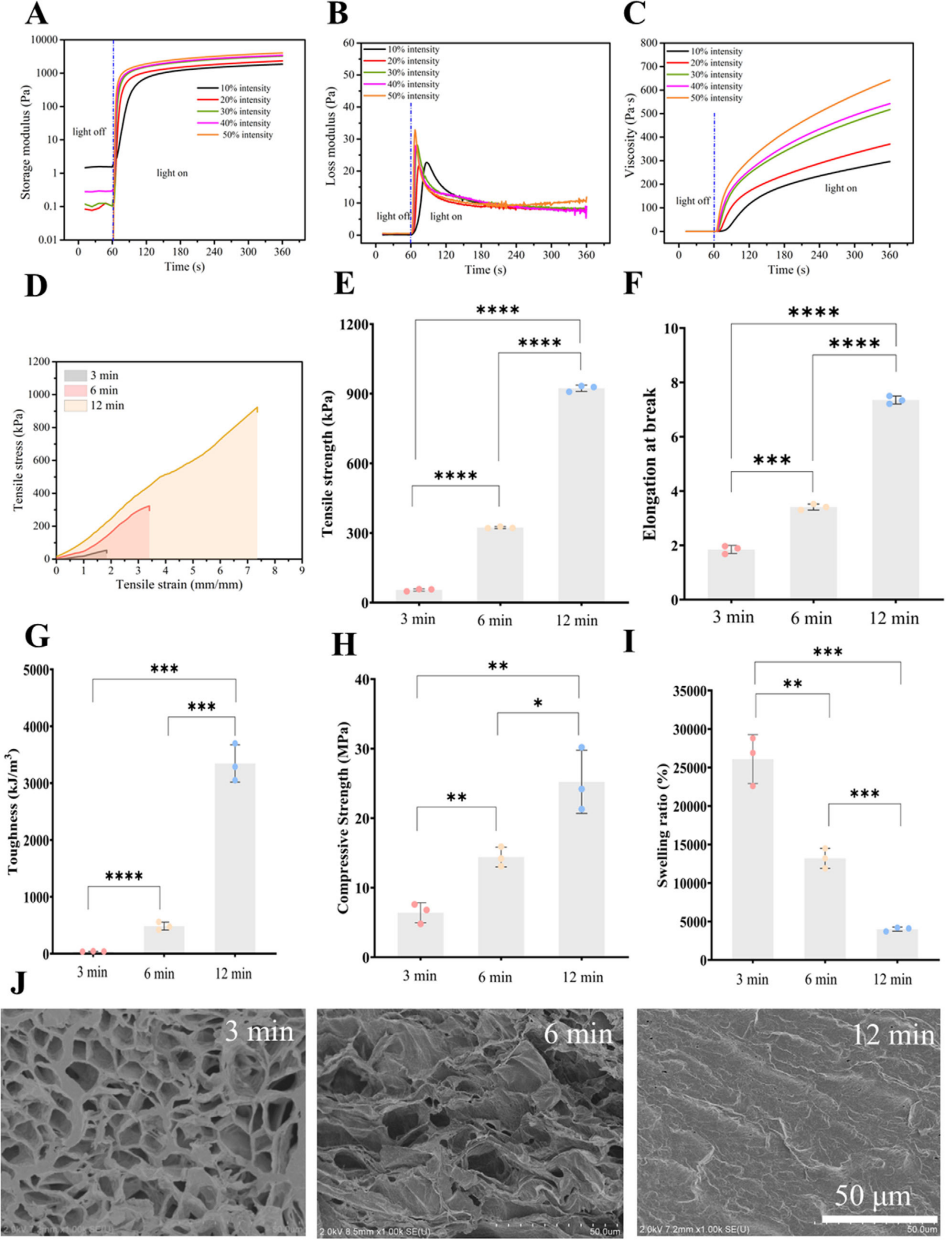
Photocuring kinetics of PVA‐SbQ hydrogels represented by A) storage modulus (G’), B) loss modulus (G’’), and C) viscosity in situ crosslinking, for all in situ crosslinking characterizations, light irradiation started at t = 60 s and ended at t = 360 s. Mechanical characteristics of PVA‐SbQ hydrogels prepared by different crosslinking durations–D) tensile stress–strain curves, E) tensile strength, F) elongation at break, G) toughness, H) compressive strength, I) swelling ratio and J) microscale structure. Error bar presented as mean ± SD, n = 3, and P‐values calculated using two‐tailed unpaired Student's t‐test assuming equal variance. ^*^ *P* ≤ 0.05, ^**^ *P* ≤ 0.01, ^*^ *P* ≤ 0.001, and ^****^ *P* ≤ 0.0001.

The unique photodimerization structure of PVA‐SbQ hydrogel not only provided the ability to be crosslinked under low‐intensity visible light emitted by a projector but also could serve as a regulator of hydrogel properties. Real‐time monitoring of hydrogel crosslinking is crucial for understanding the kinetics of gelation and the evolution of mechanical properties during the crosslinking process.^[^ ^33^ ^]^ Observing changes in storage and loss moduli over time allows valuable insights into the rate and extent of crosslinking formation, thereby facilitating the tailoring of hydrogel properties. During the real‐time monitoring of hydrogel crosslinking, measurements of the storage modulus (G′), loss modulus (G″), and viscosity of PVA‐SbQ hydrogels were recorded over time (**Figure** **2**A–C). These moduli represented the ability of PVA‐SbQ hydrogels to store and dissipate energy, respectively, under applied stress. In the initial stages, the storage modulus and viscosity remained relatively low as the hydrogel precursor solution lacked a well‐defined network structure. However, as crosslinking progresses, the storage modulus and viscosity gradually increase, indicating the formation of a more robust network within the hydrogel. Concurrently, the loss modulus also increased initially as the precursor solution underwent viscosity changes due to crosslinking reactions. As the crosslinking reaction continued, the storage modulus continued to rise, reaching a plateau once the network structure was fully formed. At this point, the hydrogel was considered to be fully crosslinked, and its mechanical properties were stable. Meanwhile, the loss modulus started to decrease until a plateau, reflecting a transition from viscous behavior to elastic behavior as the hydrogel solidified. Furthermore, by increasing the intensity of light exposure, the crosslinking process of hydrogels could be further accelerated. The increased light intensity promoted a larger number of photocrosslinking reactions occurring simultaneously, leading to quicker formation of crosslinking within the hydrogel network. As a result, the enhanced gelation kinetics reduced the overall time required for the hydrogel to achieve the expected storage modulus (G′), loss modulus (G″), and viscosity.

Rheological measurements (Figure 2A–C) were performed using the built‐in UV light source of the rheometer, which includes a quartz plate and integrated optical module. Although the printing process employed a visible‐light projector, direct integration of this light source into the rheometer was not feasible, as it would require replacing or extensively modifying the rheometer's built‐in and costly UV illumination system to accommodate visible‐light projection. To address this discrepancy, we conducted additional gelation tests under different UV light intensities to simulate varying irradiation conditions, including those more comparable to the projector light (from 10% to 50% intensity). These results allowed us to evaluate the sensitivity of gelation kinetics to light intensity and confirmed that the photoinitiated crosslinking behavior of the PVA‐SbQ system remained robust across a range of conditions. It is important to note that while UV and visible light differ in wavelength and intensity, both can initiate the same fundamental photodimerization mechanism of the SbQ groups. Therefore, all systems ultimately undergo a sol‐gel transition, and the overall trend in rheological behavior during gelation remains broadly consistent, regardless of the specific light source used. Although the gelation time varied slightly with light source intensity, the final network properties remained similar, supporting the reliability of the rheological assessment in representing the photo‐crosslinking behavior of materials. Despite this difference, the real‐time characterization of storage modulus (G′), loss modulus (G''), and viscosity of PVA‐SbQ offered valuable insights into achieving controllable hydrogel properties when utilizing projectors for hydrogel preparation. The controllability of hydrogel properties was further demonstrated through the crosslinking process using projectors. Although a wide range of crosslinking durations was investigated (Table S1, Supporting Information), 3, 6, and 12 min were selected for detailed analysis to ensure clarity in data presentation. These time points increase by a factor of two, providing a logical temporal progression. They also correspond to distinct stages in hydrogel development, with significant differences in mechanical properties and swelling behavior. Additional data covering other time points are summarized in Table S1 (Supporting Information). Hydrogels projected for three, six, and 12 min by a home projector exhibited significant variations in key mechanical properties, encompassing tensile strength (Figure 2D,E), elongation at break (Figure 2F), toughness (Figure 2G), compressive strength (Figure 2H), swelling ratio (Figure 2I), and morphology (Figure 2J). With the increase in crosslinking time, a noticeable trend emerged wherein hydrogels displayed enhanced mechanical properties, including increased tensile strength and compressive strength, as well as improved toughness and elongation at break. Moreover, the swelling ratio tended to decrease with longer crosslinking durations, indicating a denser network structure. These changes in properties also corresponded to alterations in the morphology of hydrogels (Figure 2J). With increased crosslinking time, the porous structure of hydrogels gradually diminished. During short crosslinking times, hydrogels exhibited a highly porous structure, resulting in relatively lower mechanical properties and higher dissolution rates. Conversely, as crosslinking time extended, the porosity of the hydrogels decreased, leading to enhanced mechanical properties and reduced swelling rates. The contrast between the swelling properties and mechanical characteristics of PVA‐SbQ crosslinked for three and 12 min was significant. After surpassing a 12 min threshold for crosslinking, the mechanical and swelling properties of the hydrogel showed no substantial alteration (Table S1, Supporting Information) due to saturation in the network crosslinking. However, prolonged exposure could lead to over‐crosslinking, potentially introducing defects during printing discussed in the following sections. As shown in Figure S3 (Supporting Information), during the printing process, some areas that were not designed to be crosslinked were also becoming crosslinked due to prolonged crosslinking time (over 12 min). Therefore, the properties of the hydrogel were compared by employing crosslinking durations of 3, 6, and 12 min. If such differences are precisely concentrated at different locations within a single hydrogel, it could significantly enhance the preparation and applications of heterogeneous hydrogels.

### 2.3 Printing Demonstration

**Figure 3:**
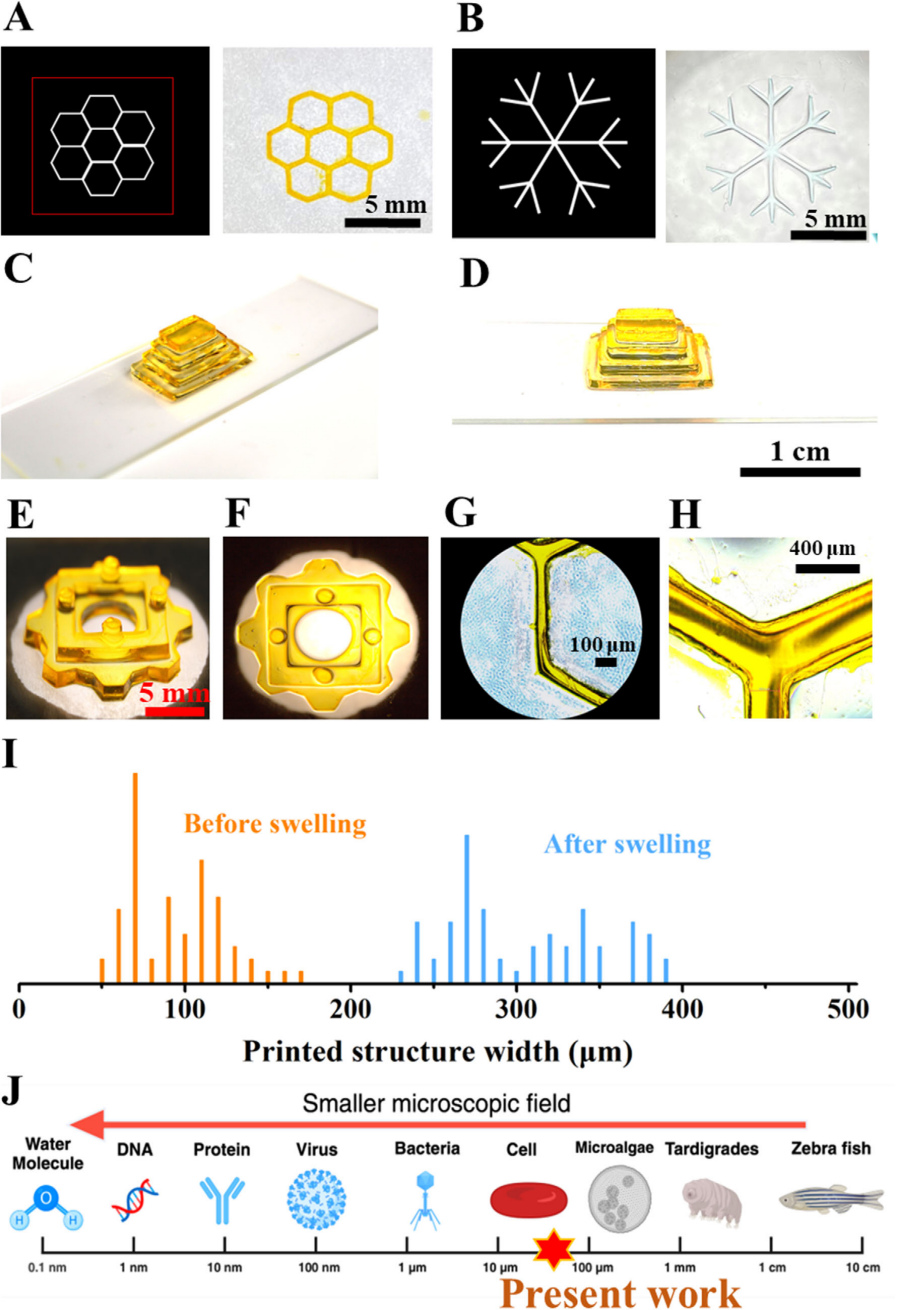
A) The designed honeycomb pattern (left) and printed PVA‐SbQ hydrogels with honeycomb structure (right). B) The designed snowflake pattern (left) and printed PVA‐SbQ hydrogels with snowflake structure (right). C) The perspective view of the printed multilayered hydrogels shaped like a pyramid. D) The front view of the printed multilayered hydrogels is shaped like a pyramid. E) The front view of printed hydrogels is shaped like a complex gear part. F) The top view of printed hydrogels is shaped like a complex gear part. G) The honeycomb pattern under a microscope. H) The swollen honeycomb pattern under a microscope. I) Line width distribution throughout the printed honeycomb scaffolds and swollen honeycomb scaffolds. J) Linear width of printed hydrogel on the microscopic scale.

The crosslinking of PVA hydrogels under the projector also enhanced their printability, enabling new possibilities for hydrogel fabrication. Specific patterns were projected onto the hydrogel precursor, prompting it to crosslink into the designated patterns. Two free‐form hydrogel structures with different complexities (snowflake and honeycomb) were printed based on the pattern of projected visible light after removing the uncrosslinked PVA‐SbQ precursor solution (**Figure** **3**A,B). Both the samples were crosslinked under visible light emitted from a projector for 6 min. In addition to 2D patterns, 3D structures shaped like a pyramid (Figure 3C,D) and complex gear parts (Figure 3E,F) could also be printed by layer‐by‐layer printing. To further examine the printed structures, the printed honeycomb pattern before and after swelling was observed and measured using a microscope. As shown in Figure 3G, by using PVA ‐SbQ bioinks with the projector printing system, high‐resolution and clear 3D scaffolds with microscale sizes could be printed rapidly. Even after swelling, the hydrogel could still maintain a stable and high‐resolution structure (Figure 3H). To further examine the printed structures, line widths were measured at different locations randomly distributed throughout the honeycomb scaffold. The line width of the printed honeycomb pattern ranged mainly between 60 and 120 µm, with 70 µm being the most prevalent. Notably, the hydrogel retained high resolution even after swelling, displaying a width distribution between 240 and 390 µm. The capability to manufacture clear structures at such dimensions had the potential to offer geometric cues to embedded cells, as demonstrated in Figure 3J.

To further highlight the advantages of our printing approach, we compared it with other commonly used hydrogel systems and printing techniques. Compared with other hydrogel systems such as GelMA, which has also been printed by Hitendra et al. using projector‐based setups, the PVA‐SbQ hydrogel offers a simpler and more efficient printing process.^[^ ^33^ ^]^ While GelMA typically requires the addition of photoinitiators and controlled exposure to UV or blue light, PVA‐SbQ can be directly crosslinked under visible light without any added initiators, greatly simplifying ink formulation and handling. Moreover, PVA‐SbQ exhibits superior printing resolution, as demonstrated in our study, making it particularly suitable for fabricating complex microscale structures. Compared to the majority of printed hydrogels (line width >100 µm), PVA‐SbQ hydrogels with intricate patterns achieved significantly higher resolution.^[^ ^33^, ^34^, ^35^ ^]^ In terms of printing speed, the visible light projection method may not achieve the same rapid throughput as the laser‐based technique we developed, which can quickly cure localized regions with high intensity in a short time.^[^ ^7^ ^]^ However, the projector‐based method offers greater flexibility in adjusting the printing process, enabling control over hydrogel properties such as crosslinking density by simply modifying crosslinking time or light intensity. Additionally, the projector‐based method is safer to operate, as it uses commercially available visible light sources without the risks associated with high‐intensity lasers. Moreover, in terms of biocompatibility, PVA‐SbQ stands out among many hydrogel inks. Unlike systems that rely on UV light and photoinitiators, which may introduce cytotoxicity or require extensive purification, PVA‐SbQ can be crosslinked using mild white light without any additional chemicals. This photoinitiator‐free and cell‐friendly crosslinking condition contributes to excellent biocompatibility, making it highly promising for applications involving live cells and in vivo use.

Although the projection‐based 3D printing setup used in this study, based on the photoreactive properties of PVA‐SbQ, offers advantages such as high resolution, simplicity, and ease of operation, it also has inherent limitations. The current system relies on a layer‐by‐layer projection approach, where each exposure is followed by manual resin replacement and re‐slicing of the model. While this method is suitable for printing small‐scale or relatively simple structures, it becomes increasingly difficult to apply when attempting to fabricate large‐scale, highly irregular tissue or organ‐like architectures. This is primarily due to the challenges associated with maintaining precise alignment across successive layers and the time‐consuming nature of the process. Therefore, the following printing in this work would focus on showcasing the fabrication of heterogeneous hydrogel structures with good fidelity, structural definition, and various applications, rather than on constructing complex organ‐level structures.

### 2.4 The Fabrication of Heterogeneous Hydrogels and Pattern Encryption

**Figure 4:**
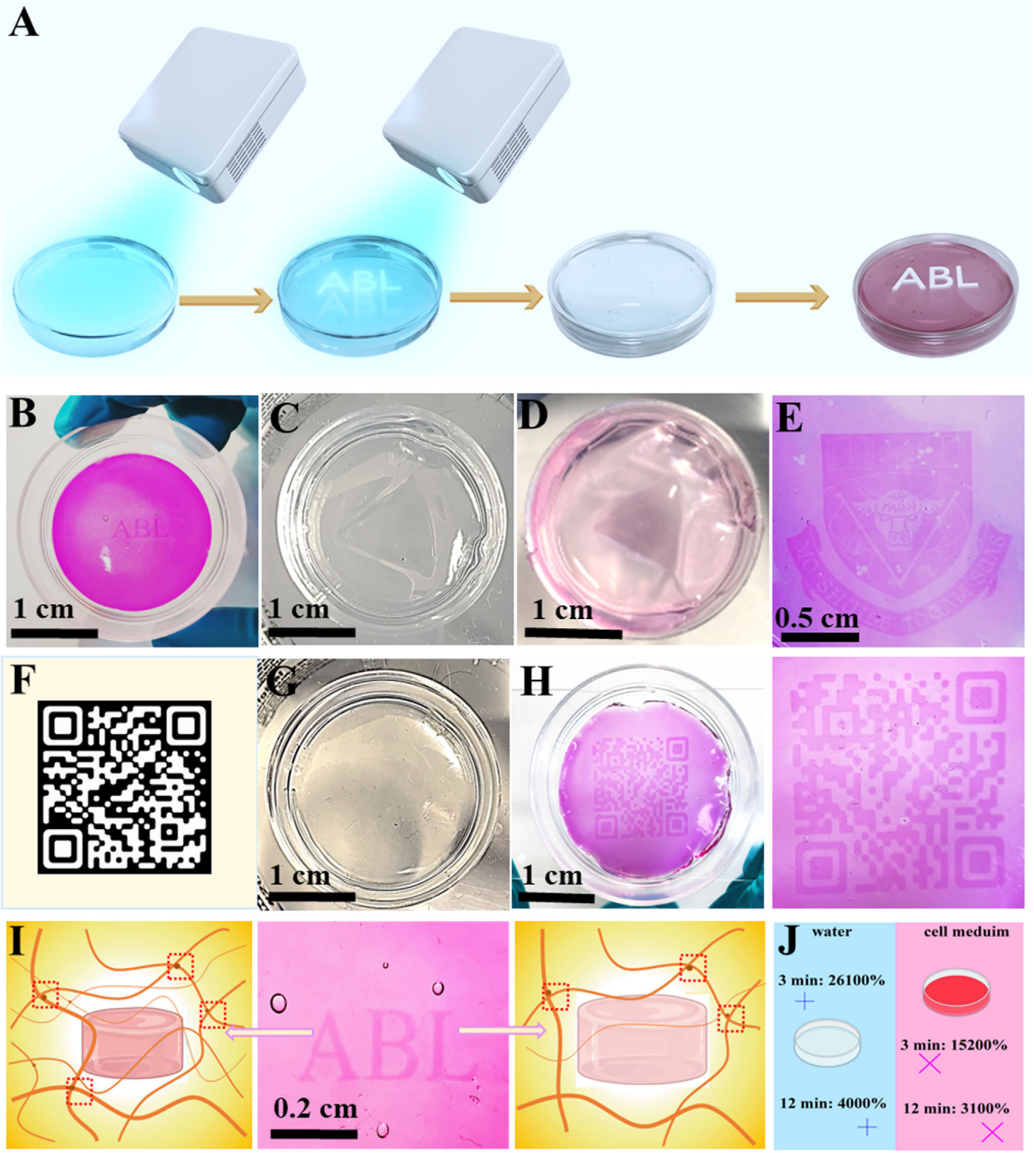
A) The method for fabricating heterogeneous PVA‐SbQ hydrogels. B) The heterogeneous PVA‐SbQ hydrogels containing the “ABL” Pattern. C) A triangle pattern is visible in water immersion. D) A triangle pattern visible in cell growth medium immersion. E) The heterogeneous PVA‐SbQ hydrogels containing a complex logo. F) The custom QR code. G) QR code invisible before swelling. H) QR code visible after swelling. I) The network difference between the pattern part and other parts. J) The difference in hydrogel swelling between water and cell growth medium.

The super high swelling ratio, photo‐tunable swelling properties, and printability of PVA‐SbQ hydrogels demonstrated in the preceding part of the text were beneficial to the fabrication of high‐resolution heterogeneous hydrogels, inspired by the heterogeneous structure of leaf veins. The high‐resolution printing of heterogeneous PVA‐SbQ hydrogels could make some invisible patterns or text on hydrogels appear after swelling by using a projector to create swelling differences at different parts of a PVA‐SbQ hydrogel. As shown in **Figures** **4**A and S4 (Supporting Information), first, the PVA‐SbQ solution was put into a petri dish and exposed to the visible light from a home projector for 3 min for crosslinking. During this process, the solution would transform into a hydrogel. Second, the light was changed to a pattern (the name of our lab: ABL) and then projected on the hydrogel so that the projected part of the hydrogel could be further crosslinked for 9 min. In this way, the total crosslinking time of the part of the PVA‐SbQ hydrogel where the ABL pattern was projected was 12 min, while the other part was crosslinked for 3 min. Third, the hydrogel that had different crosslinking times at different parts was obtained after stopping projection. The additional crosslinking time did not make the ABL pattern visible on the hydrogel. There was no difference in the surface appearance. Lastly, the hydrogel was immersed in the cell growth medium (Dulbecco's Modified Eagle Medium, DMEM). After swelling, the hydrogels could show the ABL pattern clearly after 8 h of immersion (Figure 4B). Before utilizing a cellular medium to render the pattern visible, water was initially tested. As shown in Figure 4C,D, the triangular pattern could be observed in both water and cell growth medium because of the significant swelling difference between hydrogel regions exposed to different crosslinking times. The swelling ratio of hydrogels in water was very high due to the presence of a large number of hydroxyl functional groups. Although the high swelling ratio benefits water absorption, excessive water absorption could destroy the pattern (Figure S5, Supporting Information). Compared to water, the cell growth medium could make the pattern clearer (Figure 4C,D). In addition to moderate swelling, the cell medium could interact and produce some aggregation with PVA‐SbQ by non‐covalent interactions, which resulted in an increase in light absorbance (Figure S6, Supporting Information). When the hydrogels were crosslinked for a short time, the interaction was stronger. This caused the background (the part crosslinked for 3 min) to be weakened and the pattern was more evident. Accordingly, the cell growth medium allowed for the clarity of more intricate patterns, including a university logo (Figure 4E). In the following experiments, the ABL and triangle pattern was replaced with a high‐resolution QR code shown in Figure 4F during the further crosslinking process. In this way, heterogeneous hydrogels could be used to store and encrypt information. For example, the QR code would be encrypted because it would not appear when the hydrogels are created (Figure 4G). However, the QR code became visible after swelling in an aqueous media so that the stored information could be retrieved (Figure 4H). The resolution of the QR code produced by SLA printing was sufficiently high for scanning using a smartphone and demonstrated its feasibility in practical applications (Movie S3, Supporting Information). This high resolution was attributed to the intrinsic precision of the projection‐based photopolymerization technique, as well as the favorable photo‐responsiveness and mechanical stability of the PVA‐SbQ hydrogel. Compared to conventional hydrogel‐based encryption systems, which typically exhibited feature line widths larger than 1 mm and were mainly limited to printing simple patterns or larger QR codes, this approach provided a significant improvement in spatial resolution.^[^ ^20^, ^36^, ^37^, ^38^ ^]^ Even after swelling, the minimum feature line width in this system remained between ≈290 and 340 µm (Figure 3I). However, it should be noted that the system had certain limitations in terms of pattern reversibility. Future work will focus on developing hydrogel systems with dynamic and reversible patterning capabilities to further expand their potential in applications such as reconfigurable information storage.

Regardless of the printed pattern that emerged, it could be attributed to the printing of heterogeneous hydrogels. This phenomenon arose from the difference in swelling extent within the PVA‐SbQ hydrogel. Specifically, the part of the hydrogel containing the ABL pattern exhibited a lower swelling ratio due to a longer crosslinking time, while the rest of the hydrogel had a higher swelling ratio owing to a shorter crosslinking time. Consequently, the variation in transparency and chain density during swelling accentuated the contrast between these two regions, rendering the pattern visible (Figure 4I). The crux of encryption lay in the significant difference in swelling between hydrogels crosslinked for 3 and 12 min, both in water and cell growth medium (Figure 4J). Additionally, this contrast needed to be achieved within a single hydrogel for pattern encryption.

### 2.5 4D Printing

In addition to pattern encryption, the shape of a heterogeneous hydrogel could be altered after swelling. As demonstrated in the context of pattern encryption, we implemented the same method to describe discrete regions with varying crosslinking times across hydrogel surfaces. However, when the design within these regions underwent changes, the application of the heterogeneous hydrogel transitioned from pattern encryption to 4D printing.

**Figure 5:**
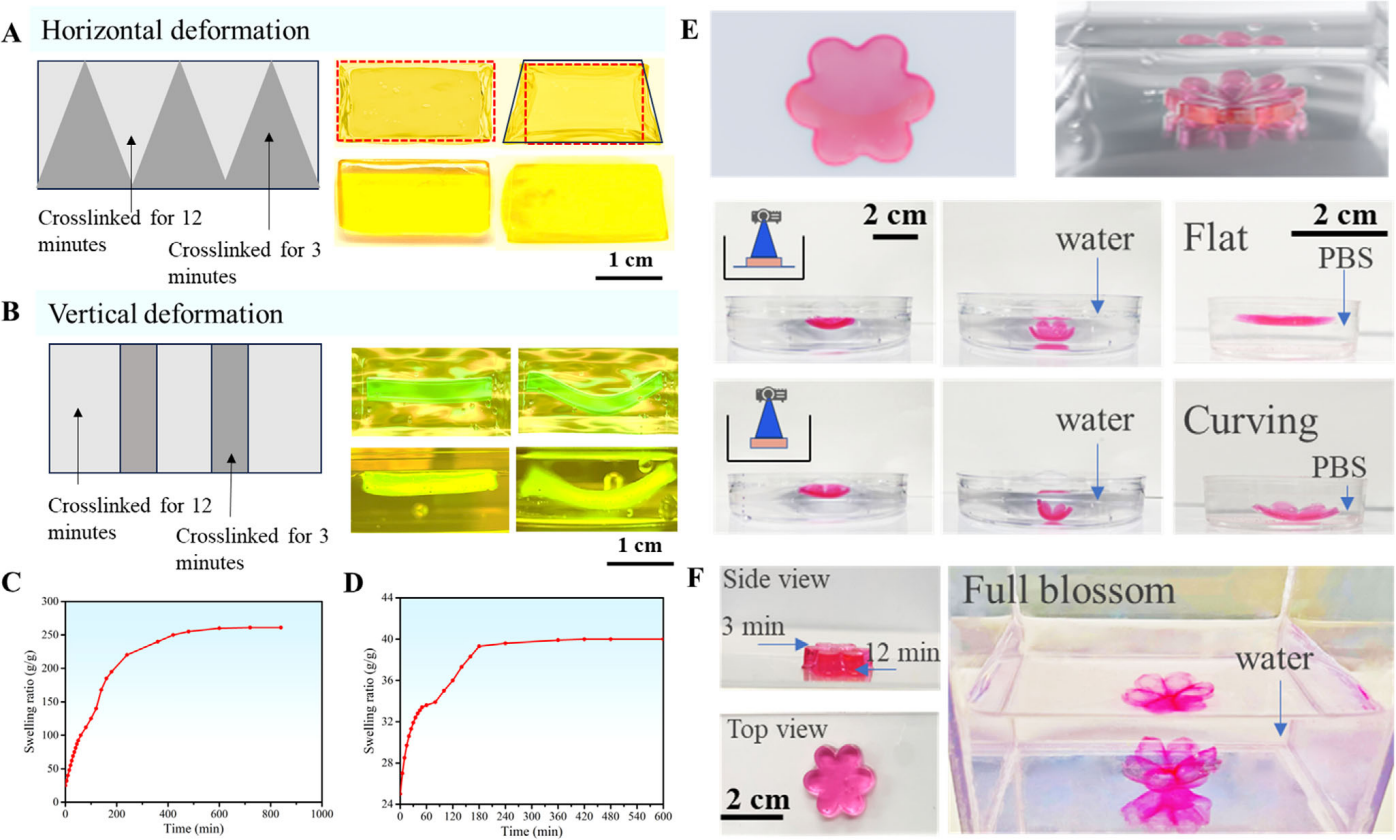
A) Horizontal deformation of heterogeneous hydrogels (From left to right: designed pattern, expected deformation, and actual deformation). B) Vertical deformation of heterogeneous hydrogels (From left to right: designed pattern, expected deformation, and actual deformation). C) Time‐dependent swelling behavior of PVA‐SbQ hydrogel crosslinked for 3 min in water. D) Time‐dependent swelling behavior of PVA‐SbQ hydrogel crosslinked for 12 min in water. E) 4D printing of single‐layer flower patterns (From top to bottom: expected deformation, and the difference in actual deformation between printing with a mirror and without a mirror. F) 4D printing of double‐layer flower patterns.

According to the design illustrated in **Figure** **5**A, the printed hydrogel exhibited a distinct rectangular pattern at the beginning. Notably, the base of the triangular area could absorb water at a higher rate due to its elevated swelling rate, resulting in increased expansion. Conversely, the opposing side experienced reduced water absorption, leading to a disparity in swelling along the horizontal axis. Consequently, the initially triangular shape underwent a morphological transformation when immersed in water, evolving into a trapezoidal configuration after 1 h. This controlled alteration in shape underscored the precision achievable through the manipulation of crosslinking times, paving the way for the development of tailored hydrogel structures with programmable functionalities. Accordingly, when exposed to specific triggers like water, tailored solvents, or moisture, the hydrogel selectively could swell in predetermined regions, inducing programmed changes in shape and morphology. This approach not only enhanced the versatility of hydrogel‐based systems but also opened up new avenues for applications in fields such as soft robotics, biomedical devices, and adaptive materials.^[^ ^39^ ^]^ Vertical changes in the hydrogel could also be achieved by fabricating sections with a shorter crosslinking time on both sides of the middle of the hydrogel, as illustrated in Figure 5B. This transformation resulted in the regular hydrogel adopting a wavy shape in 30 min. These simple deformation patterns served as foundational elements in the study of heterogeneous hydrogel deformation. Overall, both horizontal and vertical deformations were caused by the differential swelling behavior of the hydrogel in water. Upon immersion, the hydrogel began to absorb water over time. The region crosslinked for 3 min swelled significantly more than the region crosslinked for 12 min, which absorbed water at a much slower rate (Figure 5C,D). Due to the relatively rapid swelling during the early stages, this difference became evident quite quickly (Figure 5C,D). As a result, the hydrogel exhibited visible shape transformations. Notably, most of the macroscopic shape changes became apparent within the first hour due to the relatively rapid water uptake during the early swelling stage, although the exact timing might have varied slightly depending on the designed geometry. For instance, the wave‐like deformation appeared earlier than the trapezoidal deformation.

Leveraging these disparities in swelling behavior, we explored more sophisticated 4D printing methodologies. We focused on simulating the dynamic blooming of a flower in aqueous environments (Figure 5E). Initially, a single layer of hydrogel was fabricated; however, inherent differences in crosslinking density between the surface and bottom layers of the hydrogel were observed because the crosslinking of the hydrogel starts on the side of incident light (Figure 1G). To address this challenge, a corrective measure was implemented by introducing a mirror beneath the precursor liquid during the printing process, ensuring uniform crosslinking throughout the hydrogel. Subsequent experiments involved immersing the printed flowers in water, where they gradually unfolded in 15 min as the hydrogel swelled. Upon transferring the flowers to a PBS solution, a reduction in hydrogel swelling was observed, resulting in the flowers reverting to their initial state after 10 min due to a lower swelling ratio in the PBS solution. Importantly, experiments conducted without the use of a mirror reveal large differences in swelling behavior between the upper and lower regions of the hydrogel. This led to more pronounced deformations in the flowers, which persisted even upon immersion in a PBS solution. In an effort to refine our 4D printing techniques, we introduced a double‐layered flower design (Figure 5F). The bottom layer was crosslinked for 12 min, while the top layer was crosslinked for 3 min. The double‐layered flowers were both crosslinked without a mirror to achieve larger deformations. This approach yielded a more intricate and hierarchical deformation pattern in the flowers post‐printing, enhancing the precision and efficacy of the process, as shown in Figure 5F (The process requires two h to complete). By precisely engineering the crosslinking extent of the heterogeneous hydrogel, designers could manipulate its swelling behavior to achieve desired shape changes. This dynamic response allowed for the creation of complex, adaptive structures that could morph, fold, or unfold in a programmable manner. 4D printing of heterogeneous hydrogel opened up new possibilities for the design and fabrication of responsive materials with applications ranging from biomedical devices to soft robotics and beyond.

To further investigate the reversibility of the shape‐morphing behavior, we conducted a series of cycling tests under different conditions. The structures with programmed internal crosslinking gradients (Figure 5A,B,F) displayed irreversible deformation behavior. Even after re‐immersion in PBS solution or heating, the original shape could not be recovered once the hydrogel reached its swelling equilibrium. This irreversibility was likely due to the permanent internal structural rearrangements induced by the gradient design. In addition, differences in the printed pattern geometry might also contribute to the loss of reversibility. These findings highlight the influence of both internal network architecture and geometric configuration on the reversibility and cycling performance of 4D‐printed hydrogel structures.

### 2.6 Cell Organization

**Figure 6:**
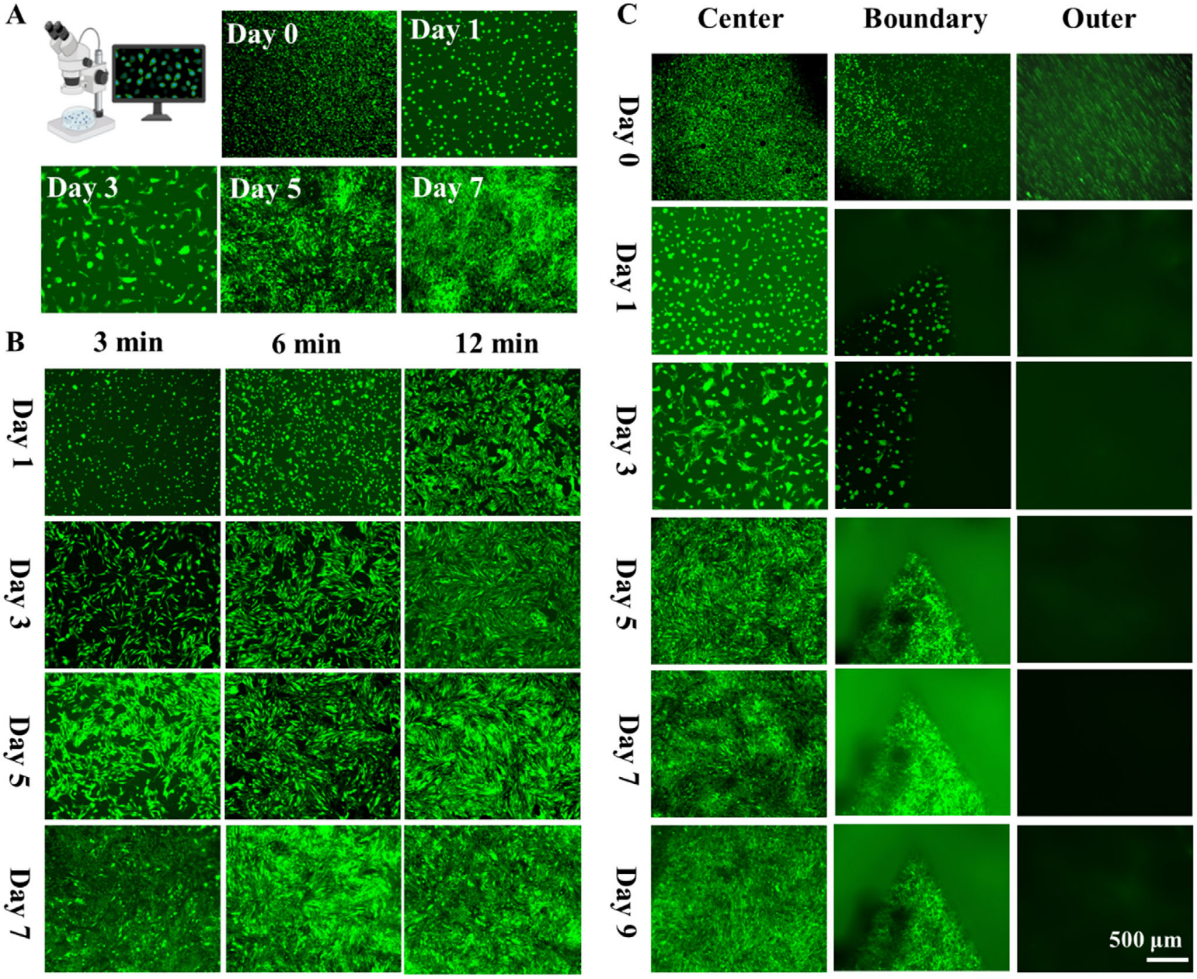
A) Micrographs of fluorescent NIH/3T3 on homogeneous PVA‐SbQ hydrogels over 7 days of culture. B) Micrographs of fluorescent NIH/3T3 on homogeneous PVA‐SbQ hydrogels crosslinked for 3, 6, and 12 min over 7 days of culture. C) Micrographs of the organization of fluorescent NIH/3T3 on heterogeneous PVA‐SbQ hydrogels over 9 days of culture. (Scale: 500µm).

Cells within tissues often need to be organized in specific spatial arrangements to perform their functions effectively.^[^ ^40^ ^]^ For example, in epithelial tissues, cells are arranged in tightly packed layers that form barriers to protect underlying tissues and organs.^[^ ^41^ ^]^ In the nervous system, neurons are organized into circuits and networks that facilitate the transmission of electrical signals.^[^ ^42^ ^]^ Cell organization is fundamental to the structure, function, and behavior of tissues and organs. The ultimate goal of biofabrication is to print tissue‐mimicking structures using biomaterials such as hydrogels.^[^ ^43^, ^44^ ^]^ Therefore, cell organization in hydrogels is a vital part of biofabrication. However, precise cell organization in bioprinted structures is quite challenging. It is because cells randomly grow in/on a uniform hydrogel. As shown in **Figure** **6**A, NIH/3T3 cells grew randomly on a single and uniform PVA‐SbQ hydrogel. Recent advances in hydrogel synthesis and microscale patterning have explored the interaction between physical cues such as stiffness and geometry in cell organization. Matrix and substrate stiffness have been reported to affect cell growth.^[^ ^45^, ^46^, ^47^ ^]^ Influenced by these studies and leveraging the controllability and printability of heterogeneous PVA‐SbQ hydrogels, we aimed to investigate the potential for controlling cell arrangement on heterogeneous hydrogels. Consequently, we initiated our exploration by preparing three hydrogels crosslinked for 3, 6, and 12 min, respectively. Different crosslinking durations resulted in different toughness and stiffness. Subsequently, we seeded NIH/3T3 cells on these hydrogels to assess the impact of toughness on cell growth and behavior. The results indicated that cells proliferated on the surface of all hydrogels, demonstrating the biocompatibility of the materials. Moreover, it was observed that the increase in crosslinking time led to enhanced cell proliferation. The longer crosslinking time resulted in better mechanical properties; therefore, the results demonstrated that a stiffer substrate was more conducive to cell growth of NIH/3T3 fibroblasts.

Based on these observations, we induced crosslinking variations within a heterogeneous hydrogel to investigate cell growth. The method for creating crosslinking variation was similar to the methods used in pattern encryption and 4D printing. Various patterns with different mechanical strengths were printed at different parts of a PVA‐SbQ hydrogel by adjusting the irradiation time at different parts of a PVA‐ SbQ hydrogel. NIH/3T3 cells preferred attaching to stiffer regions; therefore, NIH/3T3 cells could be organized in designated regions by using a projector to control the mechanical strength of different areas of a PVA‐SbQ hydrogel. As shown in Figure S7 (Supporting Information), first, the PVA‐SbQ solution was put into a petri dish and exposed to the visible light of the SLA printer for 3 min for crosslink. Second, the light was changed to a triangular pattern and then projected on the hydrogels for further crosslinking. Lastly, NIH/3T3 mouse fibroblasts were cultured on the surface of the PVA‐SbQ hydrogel. The results showed that NIH/ 3T3 mouse fibroblasts only grew at the triangular part of the PVA‐SbQ hydrogel over 9 days (Figure 3C). It was because the part of the PVA‐SbQ hydrogel where the triangular pattern was located was stiffer due to longer crosslinking time, while the other part was soft due to short crosslinking time (Figure 2G; Figure S8, Supporting Information). Usually, NIH/3T3 mouse fibroblasts grow randomly on the surface of PVA‐SbQ hydrogels when hydrogels are homogeneous (Figure 6A). However, NIH/3T3 mouse fibroblasts grew on the stiffer triangular pattern when a significant stiffness difference was created between different parts of PVA‐SbQ hydrogels by adjusting the projection time and designing the projected pattern (Movie S4, Supporting Information). On the following days, the cells also only appeared at the stiffer center part of the PVA‐SbQ hydrogel, where the triangular pattern was projected, and the cell population increased observably (Figure 6C). There were no cells located at the softer outer regions of the PVA‐SbQ hydrogel that were not further crosslinked. ≈100% of the cells remained within the stiffer triangular regions, indicating a strong preference for the more crosslinked areas. Especially at the boundary of the patterns, the phenomenon was more pronounced, and the cells were preferably located at the stiffer triangular pattern and would not cross the pattern boundary. In addition to the triangular pattern, the stiffer circular pattern was designed and printed on the surface of hydrogels. The same results were demonstrated (Figure S9, Supporting Information). The cell organization resulted from the different mechanical strengths of different parts generated by different crosslinking times. Our hypothesis posited that cell pattern formation arose from the selection of cell‐substrate interaction preferences. Under conditions where cells lacked choices, they settled uniformly on the hydrogel substrate. Conversely, when substantial mechanical differences existed on the surface of the substrate, cells tended to proliferate in locations with higher stiffness, leading to the formation of distinct cell patterns. In addition, the swelling stability of the hydrogel may influence not only cell cultivation but also its performance in other biomedical applications. In this study, the hydrogel demonstrated excellent swelling stability under physiological conditions (37 °C in PBS). Specifically, the swelling ratio reached equilibrium on the first day, with values of ≈11 600% for the hydrogel crosslinked for 3 min and ≈3000% for that crosslinked for 12 min. These values remained nearly unchanged over the subsequent 14 days. This stable swelling behavior provided a consistent and reliable microenvironment throughout the culture period, thereby minimizing any interference with cell behavior and spatial organization. As such, the potential influence of hydrogel swelling on the cellular arrangement process in the days following seeding could be considered negligible. Therefore, we could utilize projection printing to regulate cell growth by precisely controlling the spatial crosslinking distribution within the PVA‐SbQ hydrogel matrix.

### 2.7 Cell Alignment

In addition to cell organization, cell alignment could also be realized using this approach. Cell alignment refers to the orientation or arrangement of cells within a tissue or substrate. This alignment can occur in various forms, such as parallel, perpendicular, or random orientations, and plays a crucial role in tissue development, function, and regeneration.^[^ ^48^ ^]^ In many tissues, such as muscles, tendons, and nerves, cells need to be aligned in a specific direction to perform their functions effectively.^[^ ^48^, ^49^ ^]^ For example, in skeletal muscle tissue, muscle fibers align parallel to each other, enabling efficient contraction and force transmission.^[^ ^50^ ^]^

**Figure 7:**
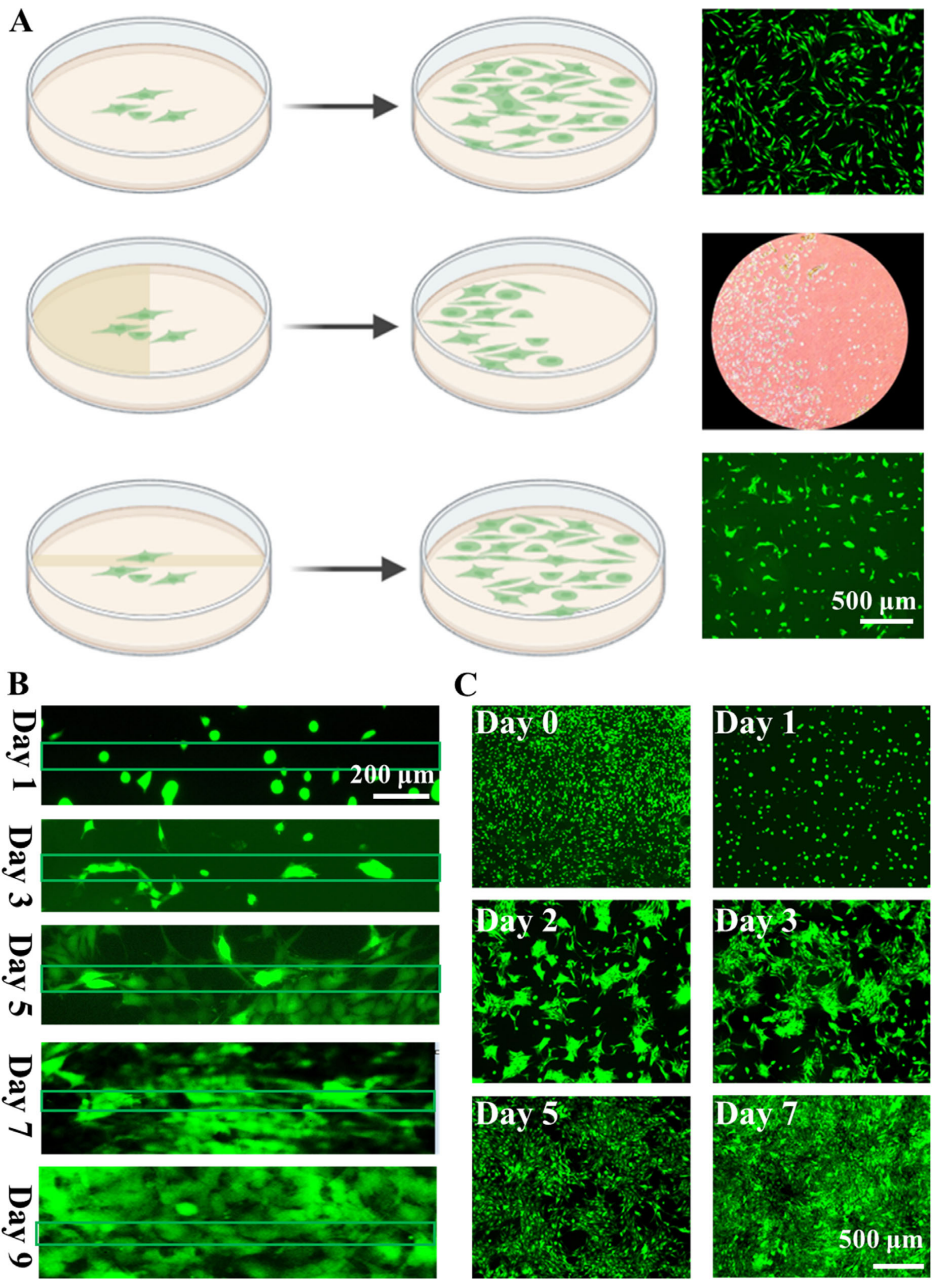
A) Three types of cell growth: random cell growth, cell organization, and cell alignment (from top to bottom) B) Micrographs of the alignment of fluorescent NIH/3T3 on homogeneous PVA‐SbQ hydrogels (a straight line) over 9 days of culture. C) Micrographs of the alignment of fluorescent NIH/3T3 on homogeneous PVA‐SbQ hydrogels (a mesh pattern) over 9 days of culture.

Using hydrogel as an example (**Figure** **7**A), when there was uniformity in the mechanical properties across the hydrogel, cells tended to grow randomly and evenly distributed. However, when a significant mechanical difference was introduced between different regions of the hydrogel, such as crosslinking one side for 12 min and the other for 3 min, cells selectively grew on the side with higher mechanical strength. As shown in Figure 7A, mouse myoblasts C2C12 (red background) were only visible on the left side. It shows that in addition to the NIH/3T3 cell line (Figure 6C), C2C12 also could be organized. Conversely, when the mechanical differences between the hydrogel and areas with longer cross‐linking times reduced, such as by creating a small region with a different crosslinking duration on the surface, cell alignment emerged (Figure 7A). This phenomenon highlighted the influence of mechanical cues on cell behavior and the potential for engineering cell alignment and patterning on hydrogel matrices. In experiments involving a 6 min crosslinked line within a 3 min crosslinked hydrogel (Figure 7B), cells within the straight‐line region (crosslinked for 6 min) exhibited parallel growth along the line. Over time, uniform alignment emerged as cell proliferation progressed, as depicted in Figure 7B. The average orientation angles of the aligned cells on Day 3, 5, 7, and 9 were 6.3°, 11.125°, 2.6°, and 3°, respectively. Similar behavior was observed when multiple lines were patterned onto the hydrogel surface (Figure S10, Supporting Information). Cells populated the entire hydrogel, with those within the grid displaying organized alignment, while those outside exhibited random growth (Figure 7C). To offer a clearer and more intuitive depiction of cell alignment, an enlarged Figure 7C with grid overlays was provided in the supporting information (Figures S11 and S12, Supporting Information). Taking Figure S12 (Supporting Information) as an example, the orientation angle of aligned cells in the mesh pattern was ≈9.5°, which was lower than the orientation angle of freely grown cells (≈45°). These findings underscored that the creation of heterogeneous hydrogels through varied cross‐linking times could not only spatially organize cell growth but also regulate cell arrangement, thereby presenting new avenues for cellular manipulation. We hypothesize that the primary factor influencing cell alignment was the reduction in the area of the stiffer regions. Cells appeared to prefer occupying these stiffer areas, thus directing their growth to maximize their presence in these regions.

## 3 Conclusion

In this study, the evolving realm of hydrogel fabrication was explored, with a focus on polyvinyl alcohol bearing styrylpyridinium groups (PVA‐SbQ) as a promising material for novel applications. The investigation into the crosslinking process of PVA‐SbQ hydrogel precursors under low light intensity from a home projector unveiled its extraordinary potential. Contrary to conventional methods requiring harmful crosslinkers and photoinitiators, PVA‐SbQ was found to circumvent oxygen inhibition and enable high‐resolution heterogeneous fabrication with tunable hydrogel properties via a radical‐free process. Furthermore, the versatility of PVA‐SbQ hydrogels in various applications, ranging from pattern encryption to 4D printing and precise cell manipulation, was showcased. Through meticulous manipulation of crosslinking time and spatial distribution, intricate patterns within heterogeneous hydrogels were demonstrated. Specifically, in pattern encryption, it was shown how hydrogels can conceal information that becomes visible only upon swelling, suggesting potential applications in secure data storage and encryption. In the domain of 4D printing, this study illustrated how PVA‐SbQ hydrogels enable shape transformations without external stimuli, offering promise in dynamic and responsive material design. Additionally, the findings underscored the ability to precisely organize and align cells within hydrogel matrices, opening avenues for advances in tissue engineering and regenerative medicine. Overall, the research highlighted the transformative impact of crosslinking time control with a home projector, offering a safer, more efficient, and highly customizable approach to fabricating high‐resolution heterogeneous hydrogel structures with tailored properties. These advancements offer the potential to propel innovations in biomedical engineering and beyond, paving the way for next‐generation biomaterials and biofabrication techniques.

## 4 Experimental Section

### Materials

Polyvinyl alcohol bearing styrylpyridinium group (PVA‐SbQ, 10g PVA‐SbQ polymer contains 4.1% mol SbQ) was purchased from Polysciences, USA. Gelatin derived from porcine skin (Type A, Bloom strength 300) was obtained from Sigma‐Aldrich, St. Louis, MO, USA. Eosin Y disodium salt (EY) was sourced from VWR, Mississauga, ON, Canada, while triethanolamine (TEOA) was purchased from MilliporeSigma, Oakville, ON, Canada. Additionally, glycidyl methacrylate (GelMA) was acquired from MilliporeSigma, Oakville, ON, Canada.

### Preparation of PVA‐SbQ HYDROGELS

The photosensitive polymer PVA‐SbQ was synthesized by grafting styrylpyridinium (SbQ) moieties onto a polyvinyl alcohol (PVA) backbone, which imparts visible‐light sensitivity through photodimerization. Given the complexity of the grafting process, a commercially available PVA‐SbQ solution (Polysciences, USA) was used as the starting material. First, A 15% (w/v) PVA‐SbQ stock solution was diluted to 4% (w/v) at room temperature. The diluted solution was centrifuged at 1000 rpm for 3 min to remove entrapped air bubbles. Second, the resulting solution was then photo crosslinked using a modified stereolithography setup based on a commercial digital light processing (DLP) projector (HD6510BD, Acer, Taipei, Taiwan), as shown in Figure 1A. A focusing lens was positioned in front of the projector to enhance resolution, and the collimated light was reflected by a mirror onto the hydrogel solution. The projector was placed 15 cm from the mirror, and the distance from the mirror to the sample surface was ≈7 cm. The projector emitted visible light in the 400–700 nm range, and the light intensity at the printing plane was measured at 80.8 mW/cm^2^ using a calibrated optical power meter (model 818P‐010‐12, Newport, Irvine, CA). The brightness of each projected pixel was controlled by computer input, producing a patterned array of bright and dark regions corresponding to areas of intended crosslinking. Finally, PVA‐SbQ hydrogels were formed by PVA‐SbQ solution after exposure for 3, 6, or 12 min, resulting in different crosslinking densities and mechanical properties.

### Preparation of GelMA Hydrogels

First, 5 g of powdered gelatin was dissolved in 50 mL of purified water. After complete dissolution at 48–50 °C, 9–10 ml of GMA was added to the gelatin solution dropwise. The solution was then maintained at 48–50 °C with constant stirring at 750 rpm for 12 h. Upon completion of the reaction, the solution was transferred into dialysis tubing (12–14 kDa) to be dialyzed against distilled water for 3 days with the water changed twice a day. After 3 days, the remaining solution was frozen and then lyophilized to obtain a foamy solid of GelMA. The lyophilized samples were stored at ‐20 °C for further use. In order to prepare GelMA hydrogels, 4% (w/v) GelMA was dissolved in PBS. An Eosin two‐component Type‐II photoinitiator was used for the crosslinking of prepared bioinks. The two‐component Type‐II photoinitiator was made up of 0.02 mM EY and 0.2% (w/v) TEOA. Finally, the GelMA solution would form hydrogels under similar conditions to the PVA‐SbQ hydrogels when exposed to the light from the projector.

### The Printing of PVA‐SbQ Hydrogels

The 4% PVA‐SbQ precursor solution was used for hydrogel printing. Two patterns with different features were printed: snowflake and honeycomb. The 3D models were created in Autodesk Fusion 360 and then sliced to obtain an image of the cross‐section as the photo pattern. Next, the PVA‐SbQ precursor solution was dispensed to form a 1 mm thick layer in a 35 mm petri dish. The photo pattern was then projected on the petri dish and allowed to photo‐crosslinking for 6 min. Next, the excess precursor solution was washed using distilled water to obtain the printed hydrogel.

The 3D printing of hydrogels shaped like pyramids and other complex gear parts followed a method akin to layer‐by‐layer additive manufacturing. Initially, the base layer was printed by a projector, serving as the foundation. Subsequently, additional layers were printed atop the base, gradually building the desired structure. To ensure structural integrity and proper crosslinking, the uncrosslinked precursor solution was removed after printing. A fresh PVA‐SbQ solution was then poured into the void left by the removed layer, allowing for the seamless continuation of the printing process. This iterative approach enabled precise control over the fabrication process, resulting in intricately designed hydrogel constructs with customizable properties.

### The Printing of Heterogeneous PVA‐SbQ Hydrogels

PVA‐SbQ solution underwent crosslinking when exposed to visible light from a projector. Initially, the entire solution was crosslinked, leading to the formation of a hydrogel. Subsequently, a designed pattern was projected onto the hydrogel, and the corresponding area underwent additional crosslinking. Multiple patterns were designed spanning different photocrosslinking durations for different purposes, such as pattern encryption, 4D printing, and cell organization and alignment.

### Fourier‐Transform Infrared Spectroscopy

The Fourier‐transform infrared spectrum of oven‐dried PVA‐SbQ solution and crosslinked PVA‐SbQ hydrogels were recorded by a Fourier‐transform infrared spectrometer (Nicolet 5700 FTIR, Thermo Fisher Scientific, USA) in a range of 300 cm^−1^ to 4000 cm^−1^.

### Optical Transmittance Tests

The transmittance of PVA‐SbQ and PVA‐SbQ/cell growth medium precursor solutions was evaluated by an ultraviolet–visible spectrophotometer (Shimadzu UV‐160 A, Japan). The transmittance of precursor solutions and hydrogels was measured in the wavelength range between 300 and 800 nm.

### Scanning Electron Microscopy Analysis

The morphologies of PVA‐SbQ hydrogels crosslinked for different durations were observed using a scanning electron microscope (Hitachi S4800, Tokyo, Japan). The hydrogel samples were prepared for imaging by freeze‐drying and then fractured in liquid nitrogen. Afterward, the fractured surfaces of hydrogels were sputtered with a 10 nm thin gold layer and observed at 5 kV in scanning electron microscopy for high image quality.

### Rheological Characterization

The rheological characteristics of PVA‐SbQ hydrogels were assessed using a rheometer under ambient conditions. A 25 mm diameter parallel plate geometry was employed with a consistent 0.5 mm gap maintained throughout the measurements. To evaluate the photocrosslinking kinetics, the bottom plate was substituted with a transparent quartz plate to enable light irradiation during the crosslinking process. During the recording of photocrosslinking kinetics, visible light with different intensities was directed onto the sample from below to initiate crosslinking. 1 min after the initiation of the experiment, visible light irradiation onto the sample was turned on and continued for a duration of 5 min. The evolution of the rheological properties of the gel during photocuring was monitored in oscillatory mode at a constant shear rate of 1% and angular frequency of 1 Hz. The storage (G’) loss modulus (G″) and viscosity of the photo‐crosslinking bioink were recorded simultaneously.

### Mechanical Properties Tests

The mechanical properties of hydrogels were evaluated by a tensile test by an Instron 5967 electronic universal testing machine. These PVA‐SbQ hydrogels crosslinked for 3, 6, 12 min were cut into dumbbell‐shaped samples with a width of 7–9 mm and a thickness of 3–5 mm for tensile tests. The tensile tests were performed using an Instron 5967 (Instron Co. Ltd.) under a tensile rate of 10 mm min^−1^ at room temperature. Compressive tests were carried out on cylindrical samples with a diameter of 8 mm and a thickness of 5 mm by an Instron 5967 tensile tester (Instron Co. Ltd) at room temperature. The compression rate was 2 mm/min. Measurements of each sample were repeated at least three times and the calculations were performed using a mean of the recorded data.

### Swelling Tests

The hydrogel samples were immersed in distilled water for 3 days at room temperature to achieve an equilibrium swelling state. In the next step, these swollen hydrogels were placed a 40 °C for 12 h oven to dry the hydrogels. The weight of swollen hydrogel and dried hydrogels was recorded. The equilibrium swelling ratio (ESR) was calculated by the following equation

(1) $$\mathrm{ESR}\%\;=\;W_{s}/W_{d}\;\times 100$$

where W_s_ and W_d_ are the weight of the swollen hydrogel and dried hydrogels at room temperature, respectively. The equilibrium swelling ratio was processed by retaining only the integer part and multiplying it by 100% for directional comparison. The swelling ratio at different time points was also calculated using this formula.

### Cell Culture

The GFP tagged NIH/3T3 murine fibroblast cell line was cultured in 75 mm^2^ plastic tissue culture flasks filled with the cell culture medium and incubated at 37 °C and 5% CO_2_. The cell culture media comprised of Dulbecco's modified Eagle's medium (Lonza, Basel, Switzerland) supplemented with 10% (v/v) heat‐inactivated fetal bovine serum (Thermo Fisher Scientific, Waltham, MA) and 1% (v/v) penicillin‐streptomycin (Sigma‐Aldrich). First, heterogeneous hydrogels were prepared in a 24‐well plate to form 1 mm thick discs and washed three times with PBS to remove the uncrosslinked precursor solution. Next, the cultured cells were collected by detaching from the tissue culture flasks using trypsin, followed by centrifugation at 1500 rpm for 3 min. The collected cells in the pellet were resuspended in the cell culture media and dispensed on the hydrogel discs. The cell seeding density was maintained at 50 000 cells/well. Additional cell culture media (500 µL) was added to the wells, and the samples were cultured at 37 °C and 5% CO_2_. On each day, the cell growth was imaged under a fluorescent microscope in the EGFP channel.

### Statistical Analysis

Continuous variables were expressed as mean ± SD, n = 3. Data sets were evaluated using a two‐tailed unpaired Student's t‐test assuming equal variance. Significance was defined as P ≤ 0.05. Statistical analysis was carried out using GraphPad Prism Version 10.3.1.

## Conflict of Interest

The authors declare no conflict of interest.

## Supporting information



Supporting Information

Supplemental Movie 1

Supplemental Movie 2

Supplemental Movie 3

Supplemental Movie 4

## Data Availability

The data that support the findings of this study are available from the corresponding author upon reasonable request.
