# Supplementary material for: High‐Resolution Heterogeneous Hydrogel Printing Using a Home Projector
Source: Small Methods. 2025 Jun 23;9(11):2500631. doi: 10.1002/smtd.202500631 (PMC12641360; doi:10.1002/smtd.202500631)
Supplement: Supplementary file 1 — Supporting Information [file SMTD-9-2500631-s003.docx]

**Supporting information**

High-Resolution Heterogeneous Hydrogel Printing Using a Home Projector

*Zhangkang Li,^a,b^ Jaemyung Shin,^b^ Kartikeya Dixit,^c^ Daichen Liu,^d^ Hongguang Zhang,^e^ Qingye Lu,^e^ Hitendra Kumar,*^,c,f^ Keekyoung Kim,***^,b,c^ Jinguang Hu***^,b,e^*

^a^ Basic Medical Research Center, Medical School of Nantong University, Co-Innovation Center of Neuroregeneration, Nantong 226001, Jiangsu Province, China

^b^ Department of Biomedical Engineering, University of Calgary, 2500 University Drive, NW, Calgary T2N 1N4, Alberta, Canada

^c^ Department of Mechanical and Manufacturing Engineering, University of Calgary, 2500 University Drive, NW, Calgary T2N 1N4, Alberta, Canada

^d^ Department of Chemical Engineering, University of Waterloo, 200 University Avenue West, Waterloo, ON, N2L 3G1 Canada

^e^ Department of Chemical and Petroleum Engineering, University of Calgary, 2500 University Drive, NW, Calgary T2N 1N4, Alberta, Canada

^f^ Department of Biosciences and Biomedical Engineering, Indian Institute of Technology Indore, Indore, Madhya Pradesh 453552, India

* Corresponding Authors: hitendra@iiti.ac.in (Hitendra Kumar), keekyoung.kim@ucalgary.ca (Keekyoung Kim); jinguang.hu@ucalgary.ca (Jinguang Hu)


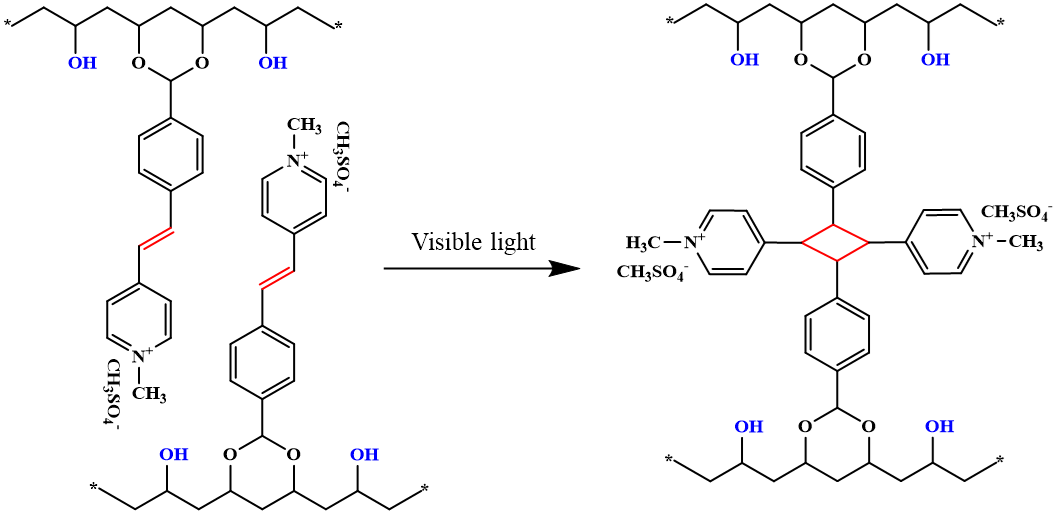


Figure S1. The photo-crosslinking mechanism of PVA-SbQ

Figure S2. The photo-crosslinking mechanism of GelMA


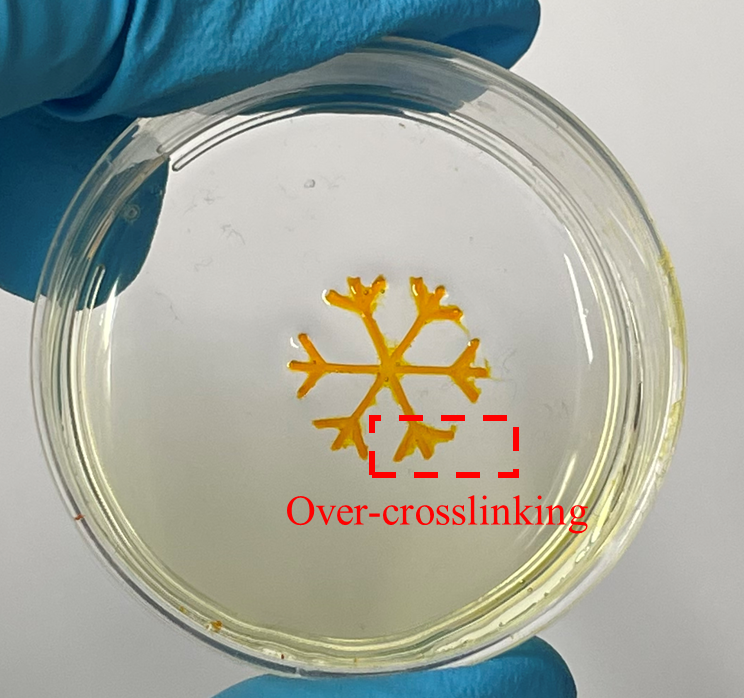


Figure S3. The over-crosslinking of printed hydrogels


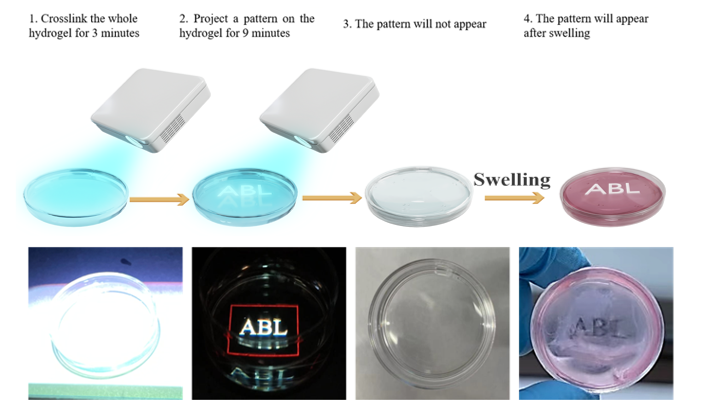


Figure S4. The method for fabricating heterogeneous PVA-SbQ hydrogels


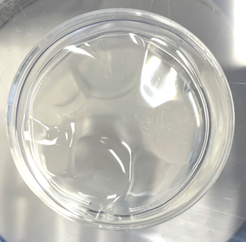


Figure S5. The excessive absorption of water





Figure S6. The light absorbance of PVA-SbQ, cell medium and PVA-SbQ/cell medium


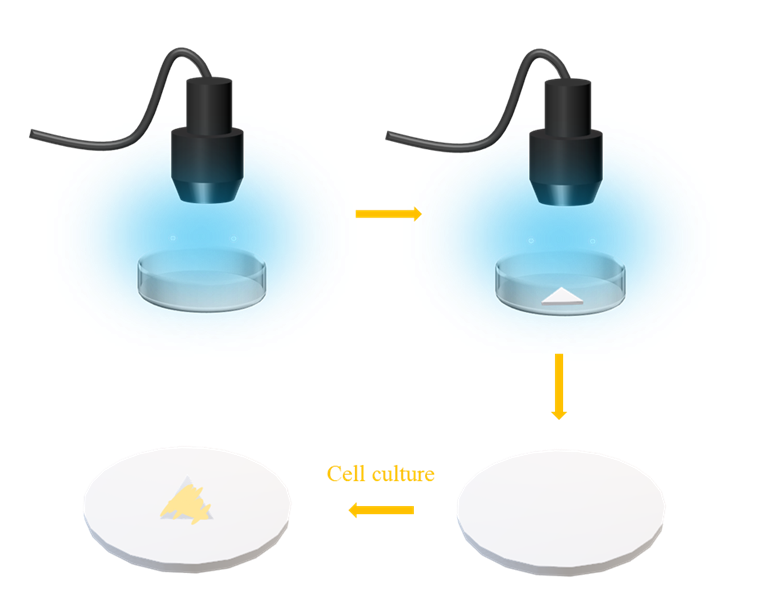


Figure S7. The method for cell organization on PVA-SbQ hydrogels


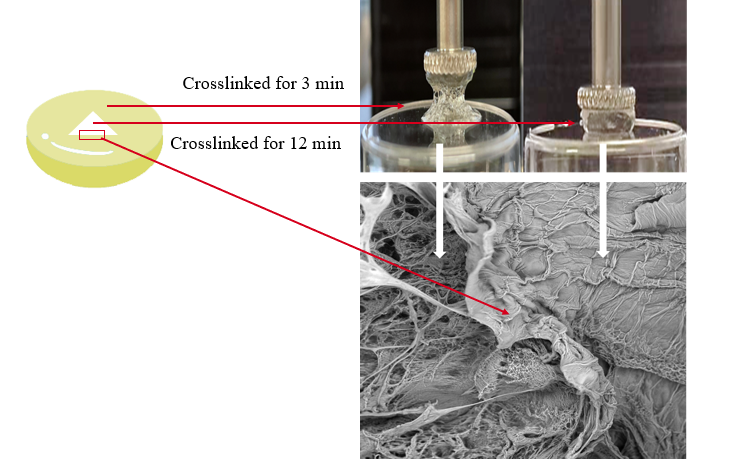


Figure S8. The difference in hydrogel properties between tough triangle patterns and other soft parts


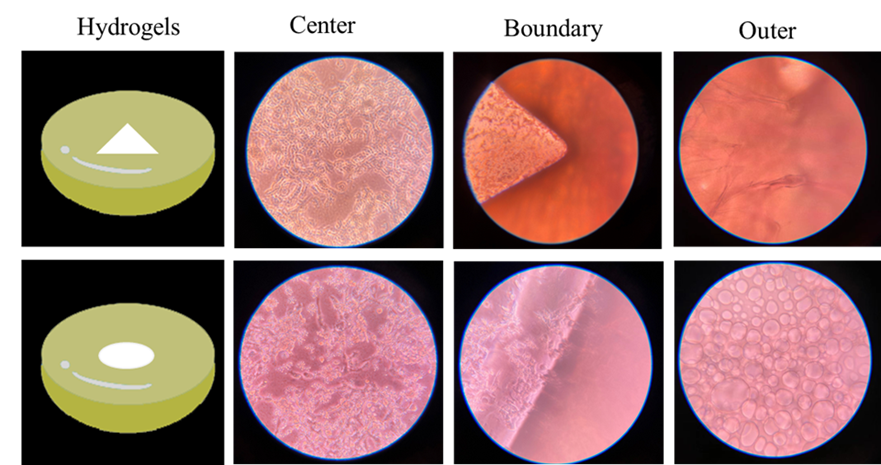


Figure S9. Cell growth on the different parts of a heterogeneous PVA-SbQ hydrogel


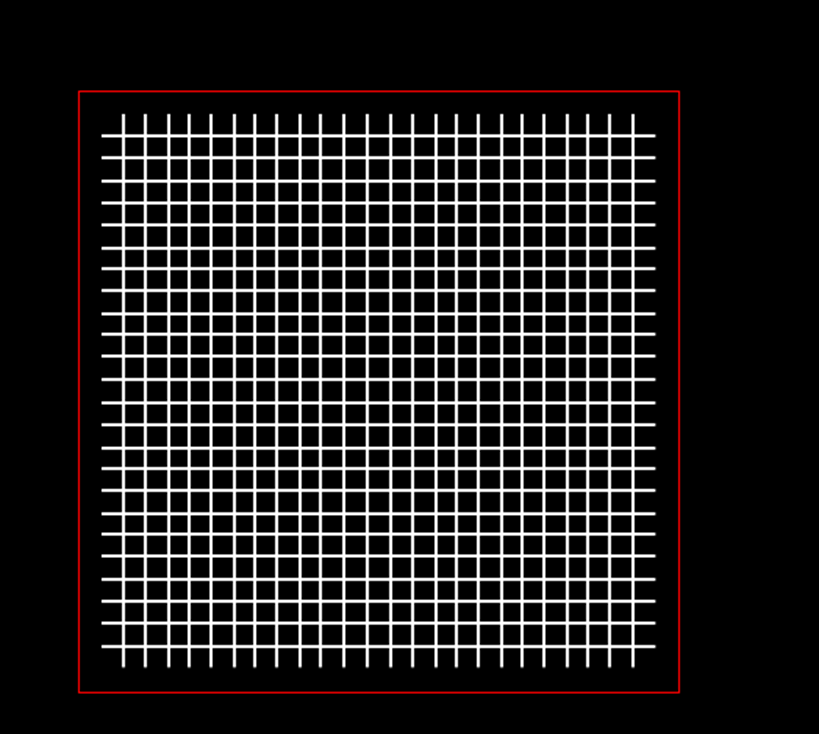


Figure S10. The designed pattern for cell alignment


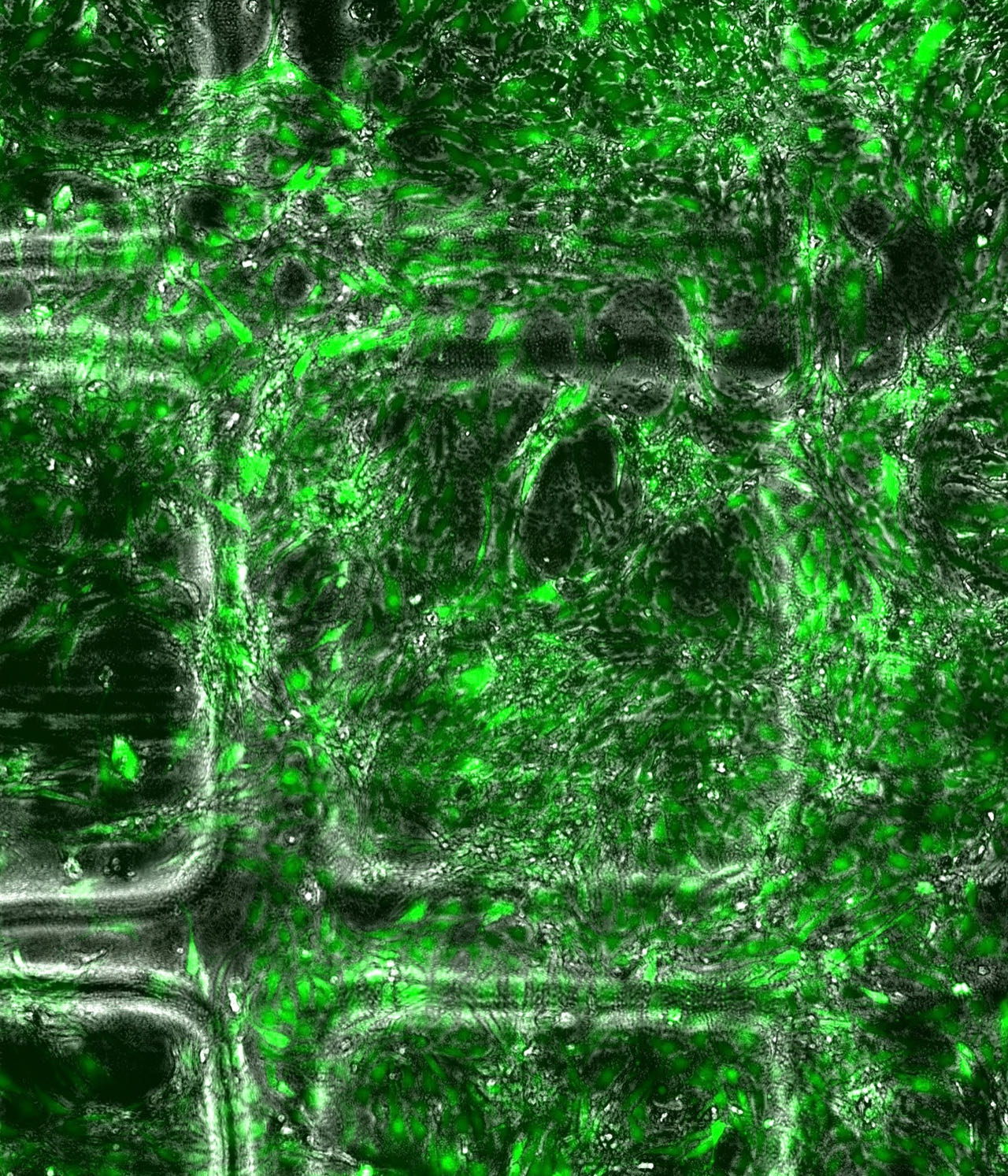


Figure S11. Micrographs of the alignment of fluorescent NIH/3T3 on homogeneous PVA-SbQ hydrogels (a mesh pattern)


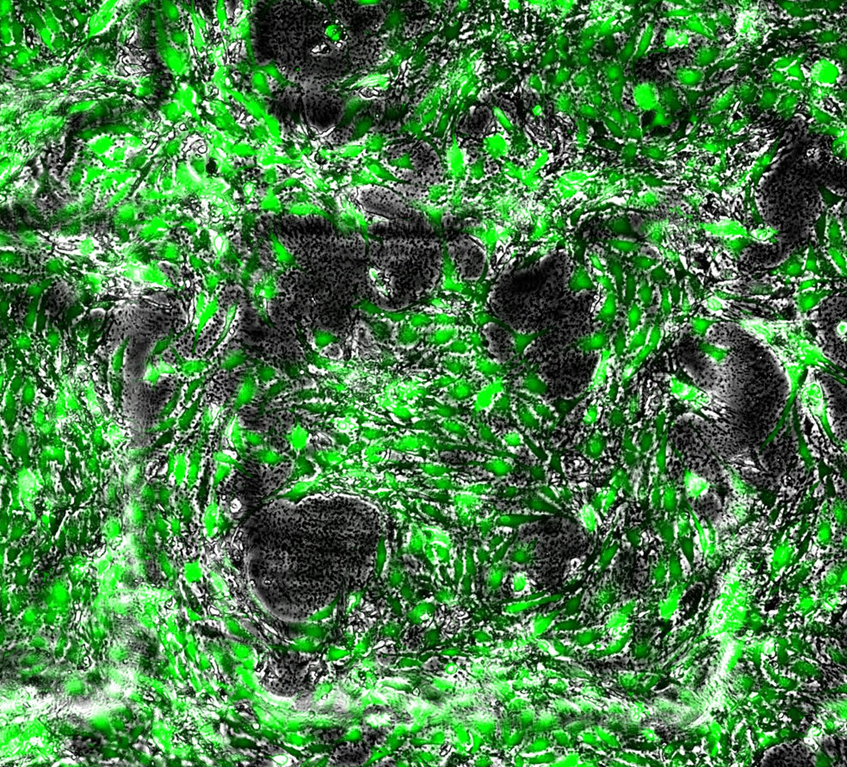


Figure S12. Other micrographs of the alignment of fluorescent NIH/3T3 on homogeneous PVA-SbQ hydrogels (a mesh pattern)

Table S1. The properties of PVA-SbQ hydrogels crosslinked for different time

| **Crosslinking Time (min)** | **Tensile Strength (kPa)** |  | | **Elongation at Break (%)** |  | **Compressive Strength (MPa)** | **Swelling Ratio (%)** |
| --- | --- | --- | --- | --- | --- | --- | --- |
| 0–3 |  | | Couldn't form hydrogels | | | | |
| 3 | 34 |  | | 184 |  | 6.4 | 26100 |
| 4 | 95 |  | | 220 |  | 8.0 | 20300 |
| 5 | 182 |  | | 279 |  | 11.0 | 16500 |
| 6 | 323 |  | | 340 |  | 14.4 | 13200 |
| 8 | 570 |  | | 493 |  | 18.2 | 8500 |
| 10 | 790 |  | | 684 |  | 22.2 | 5800 |
| 12 | 924 |  | | 735 |  | 25.2 | 4000 |
| 15 | 948 |  | | 746 |  | 27.0 | 3900 |
| 18 | 967 |  | | 752 |  | 27.5 | 3800 |

Movie S1. The crosslinking direction of GelMA

Movie S2. The crosslinking direction of PVA-SbQ

Movie S3. The scan of the QR code

Movie S4. Micrographs of the organization fluorescent NIH/3T3 on heterogeneous PVA-SbQ hydrogels
